# Characteristics of inpatients in dialectical behaviour therapy modified for a resource-limited setting

**DOI:** 10.4102/sajpsychiatry.v28i0.1701

**Published:** 2022-01-28

**Authors:** Petrus J. Steyn, Liezl Koen, Lucy Jarvis

**Affiliations:** 1 Department of Psychiatry, Faculty of Medicine and Health Sciences, Stellenbosch University, Cape Town, South Africa

**Keywords:** dialectical behaviour therapy, resource-limited settings, emotional dysregulation, borderline personality disorder, transdiagnostic

## Abstract

**Background:**

Emotional dysregulation in psychiatric disorders contributes to morbidity, mortality and healthcare costs. Dialectical behaviour therapy (DBT) is effective in addressing this, but is complex and costly to implement. Recent literature indicates that DBT can be modified for use in resource-limited settings, but little is known about its implementation in African settings.

**Aim:**

To describe the demographic and clinical characteristics of participants in a modified DBT-ST (skills training) programme at a South African psychiatric hospital.

**Setting:**

The study was conducted at Stikland Hospital, a public psychiatric hospital in the Western Cape province, South Africa.

**Methods:**

A retrospective, cross-sectional chart review of patients included in a modified inpatient DBT-ST programme between 30 June 2014 and 30 June 2019 was conducted. Descriptive analyses were performed on the data both as a complete set and after division into several subgroups.

**Results:**

We included 349 records. Two-thirds of the patients completed the programme. Major depressive disorder, borderline personality disorder and substance use disorder were the most prevalent diagnoses. Most patients had psychiatric comorbidities. A total of 90.61% (*n* = 309) of the patients were exposed to at least one traumatic event and three-quarters (*n* = 261) had attempted suicide at least once before.

**Conclusions:**

The demographics of our sample did not differ markedly from the international literature. Rather, what stood out was that modified DBT-ST could be a choice in resource-limited settings for a diagnostically heterogeneous group that displayed significant clinical complexity and high levels of emotional dysregulation. Our findings might suggest that the intervention was well tolerated and possibly most appropriately delivered at the first admission, although further research is required.

## Introduction

Dialectical behaviour therapy (DBT) is an effective treatment for borderline personality disorder (BPD). This is possibly mediated by its effectiveness in addressing emotional dysregulation, which indicates a deficiency in skills to manage emotional responses or using maladaptive strategies to do so.^1,2^ Whilst commonly observed in BPD, it is also associated with other mental illnesses, including bipolar mood disorder, chronic pain and eating disorders.^3,4,5,6^ Improvement of emotional regulation has been shown to lower the severity of substance use disorders (SUDs).^7^ Emotional dysregulation could therefore be a prime treatment target in mental illness, which has increased interest in DBT for trans-diagnostic application.

Standard DBT is a 12-month outpatient programme consisting of skills training groups (DBT-ST), individual psychotherapy (DBT-IT), between-session telephonic coaching and weekly therapist team meetings for supervision and support.^1,8,9,10,11,12^ It represents a complex and potentially costly intervention despite proven efficacy.

However, adding only DBT-ST to treatment as usual protocol reduces individual psychiatric morbidity, emergency centre visits and hospitalisations.^8,13^ The DBT-ST can also be adapted for resource-limited settings or added to treatment as usual protocols.^11,13^ Recent studies further show that DBT-ST can be delivered successfully by nurses and community workers.^14,15^ Evidence from small studies in rural Nepal with paraprofessional providers suggest that it can be adapted to local conditions without compromising efficacy.^16^

Dialectical behaviour therapy might therefore be modifiable for implementation by less highly skilled facilitators whilst still remaining effective. This would have potential benefit in low- and middle-income countries (LMICs), which often have high burdens of mental illness but lack resources to meet these demands.^17^ About 75% of suicide deaths occur in LMICs, however, less than 25% of suicidal people receive any treatment in these countries.^16,18^ The situation is especially dire in Africa, which has 0.9 mental healthcare workers per 100 000 of the population, 10 times lower than the global average.^19^ In South Africa, nearly one in three people will suffer from mental illness in their lifetime, with several of these diagnoses being potentially amenable to DBT intervention, but access to mental healthcare is similarly constrained.^20^

Dialectical behaviour therapy-skills training might help to address this gap through its trans-diagnostic applicability for conditions characterised by emotional dysregulation.^2^ As a group-based therapy, it might increase cost-effectiveness to healthcare systems.^10^

Very limited data are available regarding the translatability of DBT to non-developed, resource-limited settings outside the United States (US).^16^ On our recent review of the literature, no studies on DBT in Africa could be found. There is a need for greater understanding of DBT in this environment before it can be recommended in the local context.

Our study aims to describe the demographic and clinical characteristics of participants in a modified DBT-ST programme at Stikland Hospital in Cape Town, South Africa, to help address this shortcoming.

## Methods

### Study design

We conducted a retrospective, cross-sectional chart review of patients included in the inpatient DBT-ST programme at Stikland Hospital with the objective of providing a descriptive summary of their demographic and clinical characteristics.

### Study setting

Stikland Hospital is a public psychiatric hospital servicing a large urban and rural population in the Western Cape province, South Africa. The inpatient DBT programme entails DBT-ST groups, facilitated by psychiatric registrars and intern clinical psychologists, which are conducted twice weekly. Every week is devoted to a single module, that is, mindfulness, interpersonal effectiveness, distress tolerance and emotional regulation. The senior psychologist running the outpatient DBT group at the hospital developed a series of notes adapted to the time-constrained delivery of DBT-ST and this forms the didactic backbone. The groups are offered to selected inpatients of Intlalo Clinic, a therapeutic unit, based on referral by the multidisciplinary team (MDT). Weekly supervision of the group of facilitators is conducted by senior clinical psychologists. No patients receive formal DBT-IT. Some patients might receive individual psychotherapy or psychopharmacological management, based on the recommendations of the MDT.

#### Referral to the programme

Intlalo Clinic accepts referrals of adults between the ages of 18 and 60 years from the Stikland Hospital drainage areas. Referrals are made from primary or general specialist care, and indications include inpatient management of treatment resistant or diagnostically complicated mood, anxiety or trauma-related disorders; diagnostic admissions for complicated clinical cases and psychosocial rehabilitation through a recovery programme for patients who have suffered a major psychiatric episode. Exclusion criteria are SUDs as the primary diagnosis, active psychotic disorders, and imminent suicide risk.

The DBT programme itself has informal referral criteria based on an initial assessment by a psychiatric registrar or intern clinical psychologist and discussion by the MDT.

### Study sample

We performed a chart review of all patients included in inpatient DBT-ST between 30 June 2014, when the programme commenced, and 30 June 2019. Convenience sampling was utilised. We aimed to include all patients who had started at least one module of the programme and for whom adequate records were available. Beyond this, no exclusion criteria were applied.

### Data collection

We extracted data from patients’ folders and from the Clinicom Application Manager, an electronic database of the Western Cape Department of Health, which maintains records of patients’ contacts with public healthcare services, including outpatient appointments and hospital admissions.

Data extraction was completed manually and assembled in Microsoft Excel by the principal investigator. Demographic data (age, gender, relationship status, living arrangements, highest level of education attained, employment and source of income) were routinely recorded by clinicians (usually in the first entry of an admission) and could be readily extracted. Conversely, the extraction of clinical data (trauma history, forensic history, previous admissions to any psychiatric unit, psychiatric diagnoses, non-suicidal self-injurious behaviours [NSSI], non-fatal suicidal behaviours [NFSB], substance use, indication for DBT, utilisation of individual psychotherapy and attendance and completion of DBT) relied on interpretation of clinical notes in some instances. Absent data points in the files were taken to indicate a negative history (i.e. no reference to tobacco smoking recorded as not smoking tobacco). In cases where a certain data set was absent from the file (e.g. no mention of NFSB in a file), this was recorded as no information and the patient was excluded from analyses of these data sets. This procedure was applied to all clinical data, with the exception of psychiatric diagnoses (all Diagnostic and Statistical Manual of Mental Disorders, 5th edition [DSM-5] diagnoses on discharge included) and previous psychiatric and non-psychiatric admissions, participation in individual psychotherapy and completion of DBT, which could be extracted from folders or from the Clinicom database.

### Data analysis

The principal investigator summarised the data using descriptive statistics. We analysed the data as a complete set and investigated several subgroups, which were divided according to gender, trauma history, history of self-harming or suicidal behaviour, diagnosis, substance use, inclusion in individual psychotherapy and completion of DBT.

### Ethical consideration

Ethical clearance for the study was obtained from the Health Research Ethics Committee of the Faculty of Medicine and Health Sciences at Stellenbosch University (HREC reference number S19/10/237) and the Western Cape Health Research Committee.

Stringent protocols were put in place to ensure the safety and confidentiality of patient records during the data collection phase and all collected data were anonymised. A waiver of informed consent was granted in view of this and the retrospective nature of the study.

### Results

We identified 355 records that met inclusion criteria for the study and excluded six (duplicate records 1.13%, *n* = 4; no records/incomplete records 0.56%, *n* = 2) to finally include 98.31% (*n* = 349) of records. The main demographic characteristics of the sample are summarised in Table 1.

**TABLE 1:** Demographic characteristics of total sample.

Demographic variables	Total sample (*n* = 349)						Female (*n* = 291)						Male (*n* = 58)					
	*n*	%	Average	Median	Range	*s.d*	*n*	%	Average	Median	Range	s.d	*n*	%	Average	Median	Range	*s.d*
**Age (years)**	-	-	34.43	34	18–59	9.94	-	-	34.48	34	18–59	10.02	-	-	34.44	33	19–56	9.31
**Education (%)**																		
Less than 12 years of formal education	82	23.50	-	-	-	-	70	24.05	-	-	-	-	12	20.69	-	-	-	-
12 years of formal education	203	58.17	-	-	-	-	169	58.08	-	-	-	-	34	58.62	-	-	-	-
Tertiary qualification	64	18.34	-	-	-	-	52	17.87	-	-	-	-	12	20.69	-	-	-	-
**Employment (%)**																		
Unemployed	216	61.89	-	-	-	-	176	60.48	-	-	-	-	39	67.24	-	-	-	-
**Regular source of income (%)**																		
Self	128	36.68	-	-	-	-	110	37.80	-	-	-	-	18	31.03	-	-	-	-
Family support	121	34.67	-	-	-	-	96	32.99	-	-	-	-	24	41.38	-	-	-	-
Benefits†	45	12.89	-	-	-	-	39	13.40	-	-	-	-	6	10.34	-	-	-	-
None	55	15.76	-	-	-	-	46	15.81	-	-	-	-	9	15.52	-	-	-	-
**Relationship status (%)**																		
Not in a relationship	214	61.32	-	-	-	-	174	59.79	-	-	-	-	39	67.24	-	-	-	-

s.d., standard deviation.

† , Including state welfare grants, Road Accident Fund payments and private unemployment benefits.

#### Psychiatric diagnoses

The distribution of the most common diagnoses is displayed in Table 2. Major depressive disorder (MDD), BPD and SUD were the most common diagnoses and only 12.61% (*n* = 44) did not have any of these three diagnoses. Of this group, bipolar spectrum disorders accounted for 56.82% (*n* = 25) of diagnoses.

**TABLE 2:** Prevalence of psychiatric diagnoses in the entire sample compared with prevalence in subgroups based on the number of comorbidities.†

Diagnoses	Prevalence in sample		Prevalence in groups according to number of comorbidities									
	*n*	%	0 comorbidities		1 comorbidity		2 comorbidities		3 comorbidities		≥ 4 comorbidities	
			*n*	%	*n*	%	*n*	%	*n*	%	*n*	%
**Total in group**	349	100.00	53	100.00	158	100.00	88	100.00	35	100.00	15	100.00
**Major depressive disorder**	200	57.31	23	43.40	90	56.96	52	59.09	22	62.86	13	86.67
**Borderline personality spectrum**	197	56.45	15	28.30	84	53.16	62	70.45	22	62.86	14	93.33
Personality disorder	135	38.68	14	26.42	59	37.34	40	45.45	17	48.57	5	33.33
Personality traits	62	17.77	1	1.89	25	15.82	22	25.00	5	14.29	9	60.00
**Substance use disorder**	134	38.40	0	0.00	42	26.58	54	61.36	25	71.43	13	86.67
**Other personality diagnoses** ‡	54	15.47	0	0.00	15	9.49	15	17.05	17	48.57	7	46.67
**Post-traumatic stress disorder**	43	12.32	1	1.89	13	8.23	15	17.05	8	22.86	6	40.00
**Bipolar mood disorder type 2**	39	11.17	3	5.66	23	14.56	8	9.09	3	8.57	2	13.33
**Generalised anxiety disorder**	32	9.17	1	1.89	7	4.43	12	13.64	5	14.29	7	46.67
**Bipolar mood disorder type 1**	29	8.31	5	9.43	13	8.23	8	9.09	2	5.71	1	6.67
**Substance induced disorders** §	26	7.45	1	1.89	9	5.70	8	9.09	6	17.14	2	13.33
**Attention deficit hyperactivity disorder**	11	3.15	0	0.00	1	0.63	4	4.55	5	14.29	1	6.67
**Schizophrenia spectrum disorders**	8	2.29	1	1.89	5	3.16	2	2.27	0	0.00	0	0.00
**Other** ¶	76	21.78	3	5.66	14	8.86	24	27.27	24	68.57	11	73.33

† , All columns sum to 100%.

‡ , Includes other cluster B traits, cluster C traits, cluster A traits, antisocial personality disorder, narcissistic personality disorder and histrionic personality disorder.

§ , Includes substance-induced mood disorder (depressive type, bipolar type, type unspecified), substance-induced anxiety disorder and substance-induced psychotic disorder.

¶ , Includes adjustment disorder (with low mood, type unspecified), eating disorders (anorexia nervosa, bulimia nervosa, type unspecified), obsessive compulsive spectrum disorders (obsessive compulsive disorder, hoarding disorder, trichotillomania), functional neurological symptom disorder, somatic symptom disorder, gender dysphoria, autism spectrum disorder, neurocognitive disorders, gambling disorder, sleep disorder (primary insomnia, type unspecified) and sexual disorder (type unspecified).

We found psychiatric comorbidity to be the rule, accounting for 84.81% (*n* = 296) of the sample with an average of 2.44 diagnoses per patient (range 1–6).

Most of the patients in the sample had previous admissions to psychiatric units (61.32%, *n* = 214), with a median of two admissions per patient (average = 2.51, range 0–33). As illustrated in Table 3, when compared with patients with no previous admissions, this group had greater clinical complexity and more repeat admissions to the unit.

**TABLE 3:** Selected characteristics of patients with any previous psychiatric admission compared with patients with no previous psychiatric admissions.

Demographic and clinical variables	Any previous psychiatric admission (*n* = 214)		No previous psychiatric admission (*n* = 135)	
	*n*	%	*n*	%
Females	176	82.24	115	85.19
Any childhood trauma	171	79.91	111	82.22
Any adult trauma	97	45.33	48	35.56
Any forensic history	47	21.96	17	12.59
NFSB 1 year prior to DBT	104	48.60	62	45.93
NSSI 1 year prior to DBT	54	25.23	24	17.78
Borderline personality spectrum diagnosis†	128	59.81	69	51.11
MDD diagnosis	127	59.35	73	54.07
SUD diagnosis	80	37.38	54	40.00
Single diagnosis	32	14.95	21	15.56
Only 1 admission to this unit	124	57.94	111	82.22
DBT on first admission	159	74.30	135	100.00

MDD, major depressive disorder; SUD, substance use disorder; NSSI, non-suicidal self-injurious behaviours; NFSB, non-fatal suicidal behaviours; DBT, dialectical behaviour therapy.

† , Includes borderline personality disorder and borderline personality traits

#### Trauma history

Trauma history was inadequately reported on in 8 charts (2.29%). The remaining sample showed a high trauma burden, with 90.62% (*n* = 309) indicating at least one traumatic event. The majority of patients had trauma events in childhood or adolescence (82.70%, *n* = 282), and the average age at the first trauma event was 8.07 years (range 1–17 years). Sexual abuse (43.70%, *n* = 149) was the most common childhood trauma event, followed by parental death or separation (32.55%, *n* = 111), emotional neglect (28.45%, *n* = 97) and physical abuse (23.46%, *n* = 80). Most of the patients experienced more than one type of trauma (65.69%, *n* = 224), with an average of 2.76 types of trauma events per patient (range 0–8).

Patients with BPD had the highest prevalence of childhood trauma (86.67%, *n* = 117), followed by those with MDD (81.50%, *n* = 163) and SUD (81.34%, *n =* 109). A smaller number of the records indicated trauma events as adults (42.52%, *n* = 145), with almost half of these being sexual abuse (19.35%, *n* = 66). On average, adult trauma events contributed 21.51% (range 0–100) of the total number of trauma events in individual patients.

#### Nonfatal suicidal behaviour and non-suicidal self-injuries

Our sample showed high rates of behaviours potentially mediated by emotional dysregulation, including NFSB (74.79%, *n* = 261) and NSSI (33.81%, *n* = 118).

The most common diagnoses amongst patients with a history of NFSB were MDD (57.09%, *n* = 149), BPD (43.68%, *n* = 114) and SUD (39.85%, *n* = 104). Those with no history of NFSB had lower rates of BPD (23.86%, *n* = 21).

**FIGURE 1:**
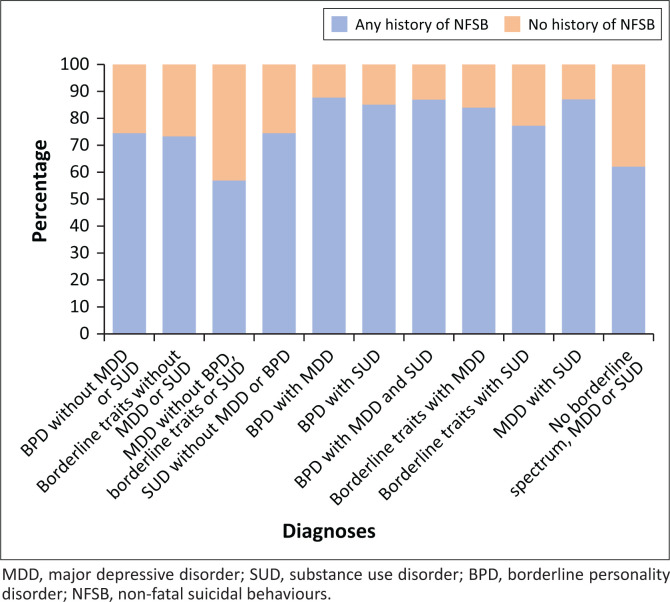
Percentage of patients with a positive and negative history of non-fatal suicidal behaviours according to diagnosis and comorbidity.

Similarly, three-quarters of patients with MDD had previous suicide attempts (74.5%, *n* = 149), but when discounting those with comorbid BPD, SUD, borderline traits or a combination of these, this number drops to 56.90% (*n* = 33). This is lower than the rate of any NFSB in patients who had no diagnosis of MDD, BPD, borderline traits or SUD (62.07%, *n* = 18). Combinations including BPD, borderline traits and SUD had lower rates of negative histories for NFSB (see Figure 1).

#### Forensic and substance histories

Table 4 demonstrates rates of substance use, substance use disorders, and a positive forensic history in the sample.

**TABLE 4:** Substance and forensic history in the total sample compared with female and male patients.

Aspects of forensic and substance history	Total sample (*n* = 349)		Female (*n* = 291)		Male (*n* = 58)	
	*n*	%	*n*	%	*n*	%
**Forensic history**						
None	285	81.66	252	86.60	33	56.90
Violent crime	16	4.58	10	3.44	6	10.34
**Substance use disorder**	134	38.40	104	35.74	29	50.00
**Substance use**						
No use	49	14.04	44	15.12	5	8.62
Alcohol	219	62.75	176	60.48	43	74.14
Nicotine	200	57.31	166	57.04	34	58.62
Cannabis	73	20.92	49	16.84	24	41.38
Medications†	76	21.78	65	22.34	11	18.97
Illegal drugs	44	12.61	30	10.31	14	24.14

† , Includes codeine containing medications and benzodiazepines.

#### Dialectical behaviour therapy participation and completion

The majority of patients were included in the DBT programme on their first admission to the unit (83.95%, *n* = 293). The reason for inclusion was mostly suspected BPD (37.25%, *n* = 130), of whom 88.46% (*n* = 115) were eventually discharged with a borderline diagnosis, defined as either BPD or borderline traits. Other inclusion indications included low mood (27.22%, *n* = 95), and emotional dysregulation, poor coping skills or suicidality (22.92%, *n* = 80).

We defined completion as attending at least six of the eight sessions, including at least one session of all modules. More than two-thirds of patients completed the programme on their first inclusion (67.34%, *n* = 235). At least half of the modules were completed by 90.26% (*n* = 315) of the patients. Non-completion occurred mostly because of scheduled discharges at a predetermined discharge date (40.35%, *n* = 46).

About half of the patients (53.30%, *n* = 186) received individual psychotherapy concurrently with participation in the DBT programme. Those who received individual psychotherapy completed DBT more often (70.97%, *n* = 132) than those who did not receive individual psychotherapy (63.19%, *n* = 103). As a rule, most patients did DBT only once, with *n* = 9 (2.58%) participating in inpatient DBT again and *n* = 8 (2.29%) participating in outpatient DBT (which resembles standard DBT more closely) either before or after the inpatient programme. The completion rate for DBT was high on second inclusion (88.89%, *n* = 8).

## Discussion

We aimed to describe the clinical and demographic characteristics of a group of patients included in an inpatient modified DBT-ST programme in a resource-limited African setting.

The demographics of our patient population did not differ significantly in terms of age, sex, level of education or relationship status from previously reported samples.^12,15,16,21^ We found a majority of female patients, as expected from the literature on gender differences in depression and BPD.^22,23^ However, we cannot exclude referral biases or confirm epidemiological factors such as sex-determined biological factors or gendered differences in help-seeking behaviour.^24^

Interestingly, studies elsewhere in Africa have shown a male preponderance in admissions, although the reported programmes were general psychiatry and included the management of psychotic disorders.^25,26^ High rates of unemployment in our sample might reflect self-selection bias because working patients might be less able to commit to long inpatient programmes.

Beyond the demographics, our key findings were that DBT-ST was utilised in the management of diverse psychopathology in patients who typically had several comorbidities and further displayed high levels of behaviours, which could be mediated by emotional dysregulation. The majority of patients completed the programme. In total, 53 different diagnoses were applied to the patients in this sample, with most patients having at least two diagnoses. To our knowledge, several of these have not been described in DBT before.

Our sample shows notable clinical complexity in terms of psychiatric comorbidities.^27,28^ Certain diagnoses were more prevalent in patients with higher comorbidity, for example, borderline diagnoses, SUD, other personality diagnoses, generalised anxiety disorder and PTSD. Borderline personality disorder is known to confer a risk of other psychiatric comorbidity.^29^ Similarly, higher SUD prevalence with higher comorbidity might be a manifestation of emotional dysregulation, shared aetiologies or reciprocal maintenance between disorders.^28^ This type of complexity might therefore be explained partially by the interplay between various diagnoses, possibly mediated by emotional dysregulation.

High rates of trauma, especially childhood trauma, might also offer some explanation of the diagnostic complexity observed. As expected, the group with borderline diagnoses had the greatest percentage of positive childhood trauma histories, but high rates of childhood trauma were found amongst other diagnoses as well. Despite the possibility of under-reporting by patients, this would fit with growing evidence of the role of childhood trauma in the development of psychiatric and other medical problems.^30,31^ Trauma causes changes in the functioning of the amygdala, hippocampus and related limbic structures which, coupled with disrupted social learning, may produce emotional dysregulation.^30^

Emotional dysregulation has high costs at individual and societal level through higher rates of substance abuse, criminality, medical service utilisation, healthcare costs, comorbid psychopathology, psychosocial morbidity, treatment resistance, poor maternal-infant attachment and suicidality.^14,16,30,31,32^ Evidence of emotional dysregulation in our sample included high rates of self-harming behaviours, substance use and positive forensic histories, which might be even more extensive than we found because of the lack of uniform documentation.

A third of the sample had a history of NSSI and three-quarters of NFSB. Combinations of diagnoses that include BPD, borderline traits or SUD were associated with more suicide attempts and self-harming. Whilst 43.10% of patients with MDD without a BPD or SUD diagnosis never attempted suicide, about 88% of those who had either or both SUD and BPD had at least one attempt. The correlation of BPD and emotional dysregulation with suicidal behaviour is well described.^3,33^ International reviews have, however, found both MDD and SUD to be common amongst suicide victims.^34^ It should be observed that whilst MDD seemingly conveyed less risk to our patients than BPD or SUD, more than half of MDD patients without either of these diagnoses still had at least one suicide attempt. Major depressive disorder is therefore not benign but impulsivity or emotional dysregulation stemming from BPD or SUD drastically increase risk.^28,33^

The high rate of previous NFSB in this sample indicates that it might be considered an independent indication for DBT-ST in this unit, although we lack a control group to conclusively infer this. This would be appropriate as DBT was originally developed to address chronic suicidality as such and would explain the diagnostic heterogeneity we found.^1,2,3,8,12^ This also highlights some of the constraints of managing these patients in resource-limited settings. In view of the wide application DBT has found in the literature, application to diverse pathology is justified, but it could also reflect limited alternative psychotherapeutic options for patients in these settings.^1,6,7,21,35,36,37,38^ Some of the possible limitations in this regard to the programme we studied include condensing DBT-ST to a 4-week programme and the diagnostic heterogeneity in groups.

An interesting question is whether this diagnostic heterogeneity is because of a lack of alternative treatments for specific diagnoses or a consequence of the informal inclusion criteria. We found that most patients were included in the programme on the basis of a suspected borderline or mood disorder diagnosis, but more than a fifth of patients were included for trans-diagnostic categories such as emotional dysregulation, poor coping skills or suicidality.

Irrespective thereof, this modified DBT-ST programme demonstrated good retention. Just over two-thirds completed DBT-ST on their first admission. The highest completion rates were in the group with three comorbidities (80.00%) and those with a single diagnosis (77.36%). The groups with better completion had higher educational attainment: a fifth of the group with single diagnoses and almost a third of those with three comorbidities had a tertiary qualification. There are no other apparent differences between the groups, and we speculate that better educated patients may fare better in these groups. This is not something that has been investigated specifically to our knowledge.

An earlier German study reported that higher rates of comorbid psychopathology and childhood abuse were amongst the factors predicting non-completion of an inpatient DBT programme, which our findings do not bear out.^39^

Tolerability of the intervention can be determined only indirectly because of lack of standardised information in the clinical records. All modules had similar completion rates and just over 90% of patients completed at least 50% of the modules. Participants included in DBT-ST for a second time completed the programme at higher rates, possibly indicating high motivation. This group was very small and results should be interpreted cautiously. Although no concrete conclusions as to acceptability can be drawn, these findings are encouraging.

Beyond high rates of comorbidity and manifestations of emotional dysregulation, there are indications in our data that repeated psychiatric admissions might not be beneficial. Those who had no previous psychiatric admissions were all included in DBT-ST on their first admission and had fewer readmissions to the unit. It has been suggested that repeated psychiatric hospitalisations can lead to unhelpful ‘psychiatrisation’ of life problems.^40^ We speculate that our findings might suggest that the first admission is the optimal timing for DBT-ST delivery so that new skills interrupt this process with the achievement of greater mastery as an outpatient. Further prospective studies are required to validate this suggestion.

At a programme level, the study exposes some of the problems of managing psychiatrically complex patients in resource-limited settings. As already mentioned, the heterogeneity of the sample could reflect a lack of resources to deliver alternative evidence-based therapies tailored to specific diagnoses. On the other hand, informal inclusion criteria allow for greater flexibility in management of complex psychopathology, especially if alternatives are limited. We also found a period during which patients were routinely discharged at a predetermined date, irrespective of whether they had completed the programme. This raises an ethical question regarding optimal utilisation of scarce resources versus optimal management of individual psychopathology. Further research into the effects of such practices would be important to inform public policy, and mental health professionals should advocate for programmes to be structured in a way that allows participants to reap maximum benefit.

## Limitations, strengths and future research

There are several important limitations to this study. Utilising descriptive statistics limits the conclusions that can be drawn. The study is an uncontrolled, retrospective chart review and records did not include objective indications of clinical severity or standardised reporting of substance use, forensic history or trauma history.

The latter point is particularly important as the risk for under-reporting could have a significant impact on findings. This bias would require prospective studies to eliminate. No findings can be made as to the efficacy of this intervention. Beyond the lack of outcome measures, treatment confounders exist. We have shown, for example, that patients who received DBT-ST concurrently to individual psychotherapy, completed the programme at a slightly higher rate than those who did not undergo individual psychotherapy. It is not clear what the significance of this is. In view of informal inclusion criteria, biases in the selection of patients for the DBT-ST programme may exist. Despite this, the flexibility of this approach in a resource-limited setting might outweigh the risk of inclusion biases in routine clinical practice. Head-to-head comparison between this programme and standard DBT programmes would be essential.

Nonetheless, the study does have several important strengths, including the size of our sample and the high inclusion rate. It is also the first study to our knowledge to look at DBT implementation in Africa and one of only a handful investigating this outside of North American or European settings. Although comorbidities have been reported in previous studies of DBT, most of the literature describes controlled trials and these comorbidities were therefore selected. This sample is one of few giving an indication of the trans-diagnostic use of DBT-ST in a real-world setting. Our findings suggest that DBT-ST groups with diagnostic heterogeneity are not inherently problematic given the high rates of completion noted.

Further research is recommended into the efficacy and tolerability of DBT-ST in our context, looking at both objective measures such as hospitalisation and subjective symptoms utilising standardised measuring instruments. Particular research attention should be given to diagnostic diversity in DBT-ST groups and comorbidity in individual patients in DBT-ST. The utility of the construct of clinical complexity should be explored in this regard. Lastly, understanding patients’ experience of DBT-ST, including its translation to and acceptability in more rural contexts, will be critical to any local recommendations for the use of DBT-ST.

## Conclusion

In describing this modified DBT-ST programme, we showed that it has been implemented in a resource-limited setting in Africa and has been utilised for patients with diverse diagnoses and high degrees of clinical complexity. There are encouraging glimpses of its potential tolerability and efficacy, although these require prospective studies in order to be verified.
